# Glutamatergic and GABAergic gene sets in attention-deficit/hyperactivity disorder: association to overlapping traits in ADHD and autism

**DOI:** 10.1038/tp.2016.273

**Published:** 2017-01-10

**Authors:** J Naaijen, J Bralten, G Poelmans, The IMAGE consortium, Stephen Faraone, Philip Asherson, Tobias Banaschewski, Jan Buitelaar, Barbara Franke, Richard P Ebstein, Michael Gill, Ana Miranda, Robert D Oades, Herbert Roeyers, Aribert Rothenberger, Joseph Sergeant, Edmund Sonuga-Barke, Richard Anney, Fernando Mulas, Hans-Christoph Steinhausen, J C Glennon, B Franke, J K Buitelaar

**Affiliations:** 1 Department of Cognitive Neuroscience, Donders Institute of Brain, Cognition and Behaviour, Radboud University Medical Center, Nijmegen, The Netherlands; 2 Department of Human Genetics, Radboud University Medical Center, Nijmegen, The Netherlands; 3 Donders Institute for Brain, Cognition and Behaviour, Radboud University, Nijmegen, The Netherlands; 4 Department of Psychiatry, Radboud University Medical Center, Nijmegen, The Netherlands; 5 Karakter Child and Adolescent Psychiatry University Center, Nijmegen, The Netherlands

## Abstract

Attention-deficit/hyperactivity disorder (ADHD) and autism spectrum disorders (ASD) often co-occur. Both are highly heritable; however, it has been difficult to discover genetic risk variants. Glutamate and GABA are main excitatory and inhibitory neurotransmitters in the brain; their balance is essential for proper brain development and functioning. In this study we investigated the role of glutamate and GABA genetics in ADHD severity, autism symptom severity and inhibitory performance, based on gene set analysis, an approach to investigate multiple genetic variants simultaneously. Common variants within glutamatergic and GABAergic genes were investigated using the MAGMA software in an ADHD case-only sample (*n*=931), in which we assessed ASD symptoms and response inhibition on a Stop task. Gene set analysis for ADHD symptom severity, divided into inattention and hyperactivity/impulsivity symptoms, autism symptom severity and inhibition were performed using principal component regression analyses. Subsequently, gene-wide association analyses were performed. The glutamate gene set showed an association with severity of hyperactivity/impulsivity (*P*=0.009), which was robust to correcting for genome-wide association levels. The GABA gene set showed nominally significant association with inhibition (*P*=0.04), but this did not survive correction for multiple comparisons. None of single gene or single variant associations was significant on their own. By analyzing multiple genetic variants within candidate gene sets together, we were able to find genetic associations supporting the involvement of excitatory and inhibitory neurotransmitter systems in ADHD and ASD symptom severity in ADHD.

## Introduction

Attention-deficit/hyperactivity disorder (ADHD) is one of the most common neurodevelopmental disorders, characterized by age-inappropriate inattentiveness and/or increased hyperactivity and impulsivity.^1^ ADHD is often accompanied by comorbidities, one of them being autism spectrum disorder (ASD). ASD is defined by impaired communication and social interaction as well as repetitive and restricted behaviors and interests.^1^ ADHD and ASD frequently co-occur, with the presence of ADHD within ASD ranging from 30 to 80%, whereas the presence of ASD in ADHD is estimated at 20–50%.^2, 3^ Both disorders are highly heritable, with estimates of 90% for ASD and ~76% for ADHD.^4, 5, 6^ In addition, ~50–70% of the contributing genetic factors are overlapping between ADHD and ASD,^7^ but also see Lee *et al.*^8^ Despite their high heritability, finding genetic risk variants for both disorders has been challenging so far. Several candidate genes have been associated with ADHD and ASD symptoms,^9, 10, 11, 12^ but genome-wide association studies have not yielded many genome-wide significance findings.^11, 12, 13^ ADHD and ASD are very heterogeneous and polygenic disorders, which may explain the difficulties in identifying the underlying genetic factors.^14, 15^ One characteristic that ADHD and ASD have in common is impairments in behavioral inhibition;^16^ for review see Wang *et al.*^13^ This overlapping trait in both disorders is linked to deficits in frontostriatal brain areas. Studying an overlapping trait in ADHD and ASD may complement the search for genetic variants involved in the disorders themselves.^17^

Glutamate is the most abundant excitatory neurotransmitter in the human brain and is involved in many neuronal functions including synaptic transmission, neuronal migration, excitability, plasticity and long-term potentiation.^18^ Because of these wide-ranging functions, altered glutamatergic neurotransmission has been implicated in many different nervous system processes.^19^ Gamma-aminobutyric acid (GABA), on the other hand, is the most abundant inhibitory neurotransmitter of the human brain involved in long-range signaling responsible for inhibition of behavior. Both neurotransmitters are involved in frontostriatal signaling, related to the dysfunctions in inhibition seen in ADHD and ASD.^20, 21^

The balance between glutamate and GABA is essential for proper brain development and functioning in these frontostriatal circuits.^22, 23^ The pathophysiology of ASD has been proposed to be characterized by a glutamate–GABA imbalance.^24^ Abnormalities in the expression of glutamate transporters, GABA-A and GABA-B receptors have been shown in post-mortem brains of patients.^25, 26^ In addition, clinical trials of glutamate receptor antagonists and GABA receptor agonists in Fragile X syndrome, which has many characteristics in common with ASD, have shown improvement of social impairments.^27^ In ADHD, an imbalance in glutamate and GABA signaling has not been reported before. Magnetic reasonance spectroscopy studies investigating GABA and glutamate in ADHD have, however, shown decreased prefrontal GABA levels in children, whereas in another study increased glutamatergic levels were found in comparable prefrontal brain areas.^28, 29^ A recent study showed a role for GABA in impulsivity and response inhibition.^30^ In addition, BOLD activity during response inhibition has been related to striatal glutamate levels, mediating the effect of dopamine synthesis on inhibition.^31^

Genes encoding glutamate and GABA receptors and transporters are candidate genes for several neuropsychiatric disorders,^32, 33, 34^ including ADHD and ASD. Deficits in inhibition can be linked to frontostriatal deficits in glutamate and GABA levels, which is consistent with findings in ADHD and ASD showing altered glutamate and GABA signaling.^35^ Genetic associations have been found for several candidate genes within the glutamatergic system. For instance, associations have been found for variation in the *GRIN2B* gene with both inattention and hyperactivity symptoms in ADHD,^36^ and both *GRIN2A* and *GRIN2B* have been associated with ASD.^37^ A genome-wide study investigating rare variants found over-representation of variants belonging to the metabotropic glutamate receptor genes in several ADHD cohorts.^38^ GABA transporter subtype 1 (*GAT1*) gene knockout mice have been shown to have decreased attention and increased impulsive behavior, relating this gene to ADHD symptomatology and difficulties in inhibiting impulses.^39^ In addition, mutations in the GABA-A receptor subunit-encoding genes *GABRQ* and *GABRA3* have been found in two different families with ASD.^40^

Both ADHD and ASD are polygenic, with multiple genetic variants with small effects assumed to have a role in a major part of the patient population. Identifying single genetic variants with a small effect size can be challenging. Considering multiple genetic variants within the same analysis can potentially increase the total explained phenotypic variance and thereby boost the power of a genetic study. Earlier studies on cognitive disorders focusing on multiple variants within the same gene or within candidate genetic pathways already showed potential for this approach.^41, 42, 43^ In addition, top findings within genome-wide association studies of psychiatric disorders have been found to converge on common underlying biological processes, suggesting that multiple genetic variants within interacting sets of genes are involved in the etiology of psychiatric disorders.^11, 12, 44, 45^

In the present study we explored, whether genes for glutamate and GABA neurotransmission are associated with ADHD and ASD traits. This was investigated by looking at the two symptom dimensions hyperactivity/impulsivity and inattention of ADHD. In addition, because of the evidence of a genetic overlap between ADHD and ASD and the glutamate–GABA imbalance hypothesis, we also investigated whether these neurotransmission gene sets moderate symptoms of ASD within an ADHD case-only sample. We used quantitative measures of ADHD and ASD symptom severity to characterize the disorders in terms of continuous distributions.^46^ Such an approach may help to better take into account the heterogeneity of the disorders as well as the extent of overlap between them.^14, 47^ Complementary, because of the common deficit of inhibitory control in ADHD and ASD and because of its regulation by frontostriatal glutamatergic and GABAergic signaling, we also investigated in a subsample whether the genes for glutamate and GABA neurotransmission are involved in inhibitory control. We made use of the stop-signal reaction time (SSRT) of a behavioral response inhibition task, which is related to excitatory and inhibitory signaling in the frontostriatal circuit. We investigated multiple genetic variants within glutamatergic and GABAergic genes simultaneously using a gene-set approach with the MAGMA software. Subsequently, we investigated gene-wide associations within this data set.

## Materials and methods

### Sample

The present study is part of the International Multi-center ADHD Genetics (IMAGE) study, an international collaborative study in seven European countries (Belgium, Germany, Ireland, Spain, Switzerland, the Netherlands and the United Kingdom) and Israel.^48, 49^ The IMAGE study was designed to identify genes that increase ADHD susceptibility. Participants were 5–17 years old and of European Caucasian descent. Exclusion criteria included an IQ below 70, the presence of a classical autism diagnosis, epilepsy, known neurological disorders and any genetic or medical disorder associated with externalizing behaviors that might mimic ADHD. Details of the IMAGE sample have been described elsewhere.^50^

### ADHD symptom severity

A semi-structured, standardized, investigator-based interview (Parental Account of Children’s Symptoms^51^) and questionnaires (parent and teacher Conners’ long-version rating scales^52^ and parent and teacher strengths and difficulties questionnaires^53^) were used to establish an ADHD diagnosis in children who were clinically diagnosed previously (see Rommelse *et al.*^54^) for the standardized algorithm that was applied to derive the DSM-IV symptoms). To investigate symptom severity, a 4-point scale was used for the questions of the subscales for inattention and hyperactivity/impulsivity of the Conners parent rating scale. Severity was defined as the summed value of the questions per subscale.

### Autism symptom severity

Autism spectrum symptoms were determined by administration of the Social Communication Questionnaire^55^ filled in by the parents. This validated questionnaire consists of 40 yes/no items determining autistic symptom severity based on the following domains: (i) reciprocal social interaction, (ii) communication and (iii) restricted, repetitive and stereotyped interests and patterns of behavior. Here, the score (ranging from 0 to 39) was used as a continuous measure of autism symptom severity. As a DSM-IV diagnosis of autistic disorder or Asperger syndrome was an exclusion criterion for the IMAGE study (see Sample), the autism symptoms could not exceed the threshold for clinical diagnosis.

### Inhibition

Response inhibition was measured using a Stop task in which participants are required to withhold their response to a stop signal as opposed to the more frequently occurring go-trials. This task was part of a larger neuropsychological assessment battery used in the Dutch part of the IMAGE study.^20^ The latency of the stop process, the SSRT, is a reliable measure of inhibition, which has shown group differences between ASD and ADHD cases compared with controls, whereas unaffected siblings showed an intermediate pattern and SSRT correlates between siblings.^20^

### Genotyping

Genome-wide genotyping of the IMAGE probands was performed as part of the Genetics Association Information Network study using the Perlegen genotyping platform, described previously.^56^ An imputation approach was used with the Hapmap II release 22 data set.^57^ The imputed data underwent quality control, in which SNPs with an imputation score lower than 0.5 and minor allele frequency lower than 0.01 were excluded. After this step, 2 182 904 SNPs across the genome were retained, excluding the X-chromosome.

Selection of the glutamate (48 genes) and GABA (36 genes) gene sets was based on Ingenuity Pathway Analysis software (http://www.ingenuity.com). This is a frequently updated genetic database for genetic pathway analysis. Ingenuity generates these ‘canonical’ pathways based on experimental evidence from the scientific literature and many other sources, including gene expression and gene annotation databases to assign genes to different groups and categories of functionally related genes. We selected genes that are known to have a distinct role in either glutamate or GABA signaling (see Supplementary Tables S1 and S2 and Supplementary Figures S1 and S2 for an overview of genes from the Ingenuity Pathway Analysis, their functions and which of those were included in the analysis). SNPs in all selected genes were selected from the quality-controlled genome-wide data set. As evidence suggests that genetic variants surrounding a gene can influence gene expression, we extracted a second set of SNPs based on the selected genes plus a 100 kilobase pair (kb) upstream and downstream flanking region.^58, 59, 60, 61^ Analyses were performed for the SNPs within all genes only (no flanking) and for the SNPs within the genes plus the 100 kb flanking region. In addition, we investigated association with genes encoding glutamate/GABA receptors and transporters only because of their most central role in neurotransmitter signaling.^62^ In this way, we wanted to check whether our results are mainly driven by these genes.

### Data analysis

Association analysis to symptom severity was performed separately for hyperactivity/impulsivity symptoms and inattention symptoms (*n*=931) and for autism symptoms (*n*=922). *Post hoc* to the analysis of association to symptoms, in a subsample (*n*=162; see Table 1) we also performed association analysis of behavioral inhibition with the glutamate and the GABA gene sets to investigate potential association of these gene sets with a common trait in ADHD and ASD. All phenotypic variables were normalized using Blom transformation (SPSS 20; SPSS, Chicago, IL, USA). Gender and age were included as covariates.

Associations were assessed using MAGMA software (Multi-marker Analysis of GenoMic Annotation, http://ctglab.nl/software/magma (ref. 63)). To account for linkage disequilibrium within our data, we used a principal components (PCs) regression model, which projects the SNP matrix for a gene on PCs and then prunes out PCs with too small eigenvalues. By default only 0.1% of the variance in the SNP data matrix is pruned away. The remaining PCs are then used as predictors for the phenotype in a linear regression model to calculate a gene-wide *P*-value.

Subsequently, we tested whether the genes in the gene set were jointly associated with the phenotype. This analysis may include a self-contained test, examining association of the set with the phenotype under a null hypothesis of no effect. However, the more valid competitive test examines whether a certain gene set of interest is more strongly associated with a phenotype than all other genes in the genome, correcting for gene size and density. In the competitive test, the effect of the gene set is compared with the background signal of all genes that are not in your gene set. Here, we only report the results of the competitive tests.

Initial testing considered gene-set associations with the phenotypes' inattention severity, hyperactivity/impulsivity severity and autistic symptom severity. The association to inhibition was performed *post hoc* in a subsample because of availability of behavioral data only for a small group.

In addition, gene-wide and single SNP associations were considered. Because of the correlations between the phenotypic variables, corrections for multiple comparisons were based on the effective number of tests calculated by using the eigenvalues of the correlation matrix. Taking the correlations into account, our number of effective tests was 2.5. For the gene sets, a *P*-value of <0.01 was considered statistically significant (*P*_corrected_=0.05/#gene-sets/#effective tests). For gene-wide association, a *P*-value of 0.0003 was considered statistically significant (*P*_corrected_=0.05/#genes/#effective tests).

## Results

Table 1 lists the general characteristics of the sample. Moderate correlations were found between the two symptom domains of ADHD (0.506, *P*<0.01) and weak correlations were found between hyperactivity/impulsivity symptom severity and autism symptom severity (0.077, *P*<0.05). No significant correlation was observed between inattention and autism symptom severity (−0.006, *P*=0.86). Similarly, no significant correlations were found between any of the clinical ADHD or autism symptom scores and the SSRT (*r*=−0.008 to 0.094, all *P-*values >0.1).

The selection of glutamate and GABA genes yielded a total of 84 genes (Table 2). Four genes positioned on the X-chromosome (*GRIA3* in the glutamate set*, GABRA3, GABRE, GABRQ* in the GABA set) were not included in the analysis because of unavailability of the X-chromosome variation in this sample. Dependent on whether flanking regions were included, the glutamatergic gene set included 42 (no SNPs in *CALML5, GRIN1, GRINA, GRM2* and *SLC7A7*) or 47 genes, consisting of 9287 and 15 466 SNPs, respectively. For GABA, the gene set without a flanking region consisted of 30 genes and 3047 SNPs (not captured were *GABARAP, GABRD* and *SLC32A21*), whereas for the 100 kb condition the set consisted of 33 genes (7534 SNPs). See Supplementary Tables S1 and S2 for SNPs per gene.

### Glutamate

The glutamate set showed association with hyperactivity/impulsivity symptom severity, as shown in Table 3. The significant competitive test (*P*=0.009) shows that the association with the glutamate gene set was stronger compared with genes that were not in the gene set, which also survived correction for multiple comparisons. No significant associations were found for autism symptom severity and inattention symptom severity (*P*=0.176 and *P*=0.144, respectively).

Single-gene analyses did not show any significant associations (Supplementary Table S5 shows the list of included genes in both gene sets and their associated *P*-values). In addition, no associations were found at the single variant level (data not shown).

The more specific association analyses that included only genes encoding for glutamate receptors and transporters showed trend-significant association with hyperactivity/impulsivity symptom severity (*P*=0.044). See also the Supplementary Table S6.

### GABA

The GABA gene set was not significantly associated with any of the symptom dimensions. Single-gene analyses also did not show any significant associations (Supplementary Table S5 shows the list of included genes in both gene sets and their association *P*-values). In addition, no associations were found at the single variant level (data not shown). Table 3 summarizes the results. Although nonsignificant when no flanking region was used, nominally significant associations were found with inhibition in the analysis including the 100 kb flanking region (*P*=0.04); however, this did not survive correction for multiple comparisons. The more specific association analyses that included only genes encoding for GABA receptors and transporters also did not demonstrate any significant associations. See also the Supplementary Table S6.

## Discussion

The present study investigated the combined effects of multiple genetic variants from glutamate and GABA gene sets with ADHD and ASD traits. The glutamate gene set was associated with hyperactivity/impulsivity symptom severity, which was robust when comparing with the rest of the genome. Although a nonsignificant competitive test could reflect a lack in power, our results indicate that GABA is not more associated with ADHD or ASD symptoms than random gene sets. GABA was, however, nominally significantly associated with inhibition. Single genes did not show significant association, suggesting that the results are because of the combined effect of genetic variants across several genes.

To the best of our knowledge, this is the first study to understand the combined effects of glutamate and GABA genes on ADHD and ASD quantitative traits. Previous investigations of other traits, which included multiple variants in one analysis, have been mainly performed based only on so-called self-contained tests. Recent research suggests that such tests might harbor the risk of type I errors because in polygenic phenotypes one will probably never find any association. MAGMA allows performance of competitive testing, in which it can be investigated whether the observed association is likely to be more specific to this gene set (a significant test) or whether its association to other genes or sets is equally possible (a nonsignificant test) by comparing it to the effect of genes that are not part of your gene set in the entire genome. A significant competitive test emphasizes the association to be specific for the tested gene set, whereas a nonsignificant test indicates that a part of the polygenic nature of the trait was captured. Self-contained tests do not take into account the overall level of association across the genome, the gene size (the number of principal components or SNPs) and the gene density. Competitive testing therefore has more merits.

The strongest finding in the current study was for the association of glutamate signaling-linked genetic variation with hyperactivity/impulsivity symptoms. This finding is in accordance with results from studies showing increased glutamate release from the prefrontal cortex of the spontaneously hypertensive rat, an animal model showing ADHD-like hyperactive behavior.^64, 65, 66^ Furthermore, glutamate has been related to self-reported impulsivity in other disorders^67^ and to impulsive behavior in healthy adolescents.^68^ Previous work in the same sample showed that the hyperactivity/impulsivity domain of ADHD was also associated with candidate genetic pathways involved in dopamine/norepinephrine and serotonin signaling,^41^ suggesting that several genetic mechanisms contribute to hyperactivity/impulsivity symptoms in ADHD cases.

In our study, we investigated the genetic underpinnings of autism symptom severity in an ADHD-only sample. ASD itself has been linked to glutamate and GABA by the excitatory/inhibitory imbalance hypothesis.^24^ Our results thus provide an interesting hypothesis for further investigating the underlying mechanism of the overlap between ADHD and ASD. Although symptoms coexist across ADHD and ASD, only weak correlations were found between hyperactivity/impulsivity symptom severity and ASD symptoms, and no correlations were found between inattention and ASD symptoms. The associations found in the current study could, therefore, not be related to the mere correlation between the studied phenotypes.

A previous study indicated excitatory/inhibitory gene expression to be disturbed in ASD, suggesting reduction of the expression of inhibitory genes to be more pronounced compared with genes related to excitation and that the imbalance was mainly due to GABA disturbances.^69^ In the present study, GABA was nominally significantly related to inhibition, which may reflect a disturbance of these inhibitory factors. Our finding was, however, limited by the small sample size used and needs replication in an independent data set before any conclusions can be drawn.

The current results support the hypothesis that glutamate and possibly also GABA are associated with ADHD and ASD traits. Unfortunately, current gene-set association analysis cannot reveal the direction of the observed effects, or whether the directions for the gene sets differ, which makes the interpretation of the findings in this study more difficult in terms of the imbalance theory for GABA and glutamate. In future studies, it would be interesting to add information on the neural mechanisms underlying inhibition by using imaging studies focusing on the frontostriatal circuitry.

Our findings should be viewed in light of certain strengths and limitations. An important strength of the current study is the combination of multiple genetic variants, which enables us to better take into account the small effect sizes for single variants and genetic heterogeneity. A second strength is the use of a competitive test that tests association of the selected gene set against the background signal of other genes. Gene-set selection is, however, limited by the fact that the currently available databases are still incomplete and/or not sufficiently annotated. For the current study, we took the approach of only including genes that could be selected by using the canonical pathway database from Ingenuity. Other approaches would have been selection based on literature on glutamatergic and GABAergic genes with a possible role in ADHD, or using proteomic studies,^70^ both of which have their drawbacks as well. Therefore, with our approach, we may have missed genes that are involved in glutamatergic and/or GABAergic signaling; however, we are confident that the genes that we included are directly involved in glutamate/GABA signaling.

As the studied sample consisted of ADHD patients only, the current results should not be viewed as related to disorder risk either for ADHD or ASD. To this end, a case–control study should first be performed. In addition, a diagnosis of autistic disorder or Asperger disorder was an exclusion criterion in the IMAGE study. We could therefore not directly compare overlapping genetic underpinnings, but could only investigate the presence of autism behavior within the ADHD sample. It would be interesting for the future to replicate these findings in an ASD sample and to include patients with ASD with comorbid ADHD. Lastly, differences in results occurred based on the flanking region we used. Although inclusion of the 100 kb flanking regions of selected genes did not seem to lead to more explained variance in most cases, the difference between using the flanking region and not using it should be highlighted. The gene sets without the flanking region capture the SNPs in the genes, whereas in the gene sets with the 100 kb flanking region there were 4487 and 6179 extra SNPs present in the GABA and glutamate set, respectively, which are not part of the gene itself (see Supplementary Tables S3 and S4 for number of SNPs per gene). In addition to including regulatory regions important for gene activity,^58, 59, 60, 61^ this flanking region may also include neighboring genes that may dilute effects of variants in the selected genes. We therefore reported both approaches in the current manuscript.

In conclusion, the current study supports the hypothesis that genes involved in glutamate neurotransmission are involved in ADHD as they were associated to hyperactivity/impulsivity severity. An overlapping trait between ADHD and ASD, altered response inhibition, may show an association with GABA; however, additional research is necessary to clarify this. In addition the present study shows that studying the aggregated effect of multiple genetic variants may overcome power problems in genetic association testing.

## Figures and Tables

**Table 1:** Demographic characteristics of the study sample

	*Value*	N
Age: y, m (s.d.)	10.9 (2.8)	946
Male: %	87.5%	946
Conners parent hyperactive/impulsive *T*-scores: mean, median (s.d.)	78,67; 80 (10,67)	931
Conners parent inattentive *T*-scores: mean, median (s.d.)	71.08; 71 (9.62)	931
Social Communications Questionnaire total scores: mean, median (s.d.)	8.33; 8 (6.11)	922
Stop-signal reaction time in milliseconds: mean, median (s.d.)	299.23; 280.08 (92.54)	162

Abbreviations: m,month; y, year.

**Table 2:** Glutamatergic and GABAergic genes selected for analysis

	*Glutamate genes*							*GABA genes*								
*CALM1*	*GRIA4*	*GRIK5*	*GRIN3B*	*GRM5*	*PICK1*	*SLC1A2*	*ABAT*	*GABRA1*	*GABRB2*	*GABRP*	*GPHN*	*UBQLN1*
*CALML5*^a^	*GRID1*	*GRIN1*^a^	*GRINA*^a^	*GRM6*	*SLC17A1*	*SLC1A3*	*ALDH5A1*	*GABRA2*	*GABRB3*	*GABRQ*^b^	*NSF*	
*CAMK4*	*GRID2*	*GRIN2A*	*GRIP1*	*GRM7*	*SLC17A2*	*SLC1A4*	*ALDH9A1*	*GABRA3*^b^	*GABRD*^a^	*GABRR1*	*SLC32A1*^a^	
*GLS*	*GRIK1*	*GRIN2B*	*GRM1*	*GRM8*	*SLC17A6*	*SLC1A6*	*DNM1*	*GABRA4*	*GABRE*^b^	*GABRR2*	*SLC6A1*	
*GRIA1*	*GRIK2*	*GRIN2C*	*GRM2*^a^	*HOMER1*	*SLC17A7*^a^	*SLC1A7*	*GABARAP*^a^	*GABRA5*	*GABRG1*	*GABRR3*	*SLC6A11*	
*GRIA2*	*GRIK3*	*GRIN2D*	*GRM3*	*HOMER2*	*SLC17A8*	*SLC38A1*	*GABBR1*	*GABRA6*	*GABRG2*	*GAD1*	*SLC6A12*	
*GRIA3*^b^	*GRIK4*	*GRIN3A*	*GRM4*	*HOMER3*	*SLC1A1*		*GABBR2*	*GABRB1*	*GABRG3*	*GAD2*	*SLC6A13*	

Abbreviations: GABA, gamma-aminobutyric acid; SNP, single-nucleotide polymorphism.

a No SNPs for analysis when no flanking region was used.

b Gene positioned on the X-chromosome.

**Table 3:** Association result *P*-values for the discovery and *post hoc* tests

	*Competitive*	
	*0* *kb*	*100* *kb*
*Glutamate*		
Autism symptom severity	0.176	0.873
Hyperactivity/impulsivity severity	**0.009**	0.263
Inattention severity	0.144	0.566
Inhibition (SSRT)	0.037	0.345

*GABA*		
Autism symptom severity	0.465	0.769
Hyperactivity/impulsivity severity	0.473	0.618
Inattention severity	0.827	0.434
Inhibition (SSRT)	0.178	*0.040*

Abbreviations: GABA, gamma-aminobutyric acid; SSRT, stop-signal reaction time.

Bold marking indicates significance after correction for multiple comparisons (Meff-corrected, adjusted *P*-value=0.01); italics show nominally significant associations.
