# Supplementary material for: Glutamatergic and GABAergic gene sets in attention-deficit/hyperactivity disorder: association to overlapping traits in ADHD and autism
Source: Transl Psychiatry. 2017 Jan 10;7(1):e999–. doi: 10.1038/tp.2016.273 (PMC5545734; doi:10.1038/tp.2016.273)
Supplement: Supplementary Material [file tp2016273x1.docx]

**Table S1.** Ingenuity Pathway Analysis (IPA) list of genes included in the glutamatergic signaling pathway, their functions and inclusion in the gene-set analysis

| **Gene** | **Function** | **Included** |
| --- | --- | --- |
| *GRIA1* | glutamate receptor 1 | Yes |
| *GRIA2* | glutamate receptor 2 | Yes |
| *GRIA3* | glutamate receptor 3 | No |
| *GRIA4* | glutamate receptor 4 | Yes |
| *GRID1* | glutamate receptor ionotropic, delta-1 precursor | Yes |
| *GRID2* | glutamate receptor ionotropic, delta-2 precursor | Yes |
| *GRIK1* | glutamate receptor ionotropic, kainate 1 precursor | Yes |
| *GRIK2* | glutamate receptor ionotropic, kainate 2 precursor | Yes |
| *GRIK3* | glutamate receptor ionotropic, kainate 3 precursor | Yes |
| *GRIK4* | glutamate receptor ionotropic, kainate 4 precursor | Yes |
| *GRIK5* | glutamate receptor ionotropic, kainate 5 precursor | Yes |
| *GRIN1* | glutamate receptor ionotropic, NMDA 1 precursor | Yes |
| *GRIN2A* | glutamate receptor ionotropic, NMDA 2A precursor | Yes |
| *GRIN2B* | glutamate receptor ionotropic, NMDA 2B precursor | Yes |
| *GRIN2C* | glutamate receptor ionotropic, NMDA 2C precursor | Yes |
| *GRIN2D* | glutamate receptor ionotropic, NMDA 2D precursor | Yes |
| *GRIN3A* | glutamate receptor ionotropic, NMDA 3A precursor | Yes |
| *GRIN3B* | glutamate receptor ionotropic, NMDA 3B precursor | Yes |
| *GRINA* | protein lifeguard 1 | Yes |
| *GRIP1* | glutamate receptor-interacting protein 1 | Yes |
| *GRM1* | metabotropic glutamate receptor 1 | Yes |
| *GRM2* | metabotropic glutamate receptor 2 | Yes |
| *GRM3* | metabotropic glutamate receptor 3 | Yes |
| *GRM4* | metabotropic glutamate receptor 4 | Yes |
| *GRM5* | metabotropic glutamate receptor 5 | Yes |
| *GRM6* | metabotropic glutamate receptor 6 | Yes |
| *GRM7* | metabotropic glutamate receptor 7 | Yes |
| *GRM8* | metabotropic glutamate receptor 8 | Yes |
| *SLC17A1* | sodium-dependent phosphate transport protein 1 | Yes |
| *SLC17A2* | sodium-dependent phosphate transport protein 3 | Yes |
| *SLC17A6* | vesicular glutamate transporter 2 | Yes |
| *SLC17A7* | vesicular glutamate transporter 1 | Yes |
| *SLC17A8* | vesicular glutamate transporter 3 | Yes |
| *SLC1A1* | excitatory amino acid transporter 3 | Yes |
| *SLC1A2* | excitatory amino acid transporter 2 | Yes |
| *SLC1A3* | excitatory amino acid transporter 1 | Yes |
| *SLC1A4* | neutral amino acid transporter A | Yes |
| *SLC1A6* | excitatory amino acid transporter 4 | Yes |
| *SLC1A7* | excitatory amino acid transporter 5 | Yes |
| *SLC38A1* | sodium-coupled neutral amino acid transporter 1 | Yes |
| *HOMER1* | homer protein homolog 1 | Yes |
| *HOMER2* | homer protein homolog 2 | Yes |
| *HOMER3* | homer protein homolog 3 | Yes |
| *GLS* | glutaminase kidney isoform, mitochondrial isoform precursor | Yes |
| *CALM1* | Calmodulin | Yes |
| *CALML5* | calmodulin-like protein 5 | Yes |
| *CAMK4* | calcium/calmodulin-dependent protein kinase type IV | Yes |
| *PICK1* | PRKCA-binding protein | Yes |
| *GNB1* | guanine nucleotide-binding protein G(I)/G(S)/G(T) subunit beta-1 | No |
| *GNB3* | guanine nucleotide-binding protein G(I)/G(S)/G(T) subunit beta-3 | No |
| *GNG11* | guanine nucleotide-binding protein G(I)/G(S)/G(O) subunit gamma-11 precursor | No |
| *GNG2* | guanine nucleotide-binding protein G(I)/G(S)/G(O) subunit gamma-2 precursor | No |
| *GNG5* | guanine nucleotide-binding protein G(I)/G(S)/G(O) subunit gamma-5 precursor | No |
| *GNG7* | guanine nucleotide-binding protein G(I)/G(S)/G(O) subunit gamma-7 precursor | No |
| *GNG10* | guanine nucleotide-binding protein G(I)/G(S)/G(O) subunit gamma-10 precursor | No |
| *GNG12* | guanine nucleotide-binding protein G(I)/G(S)/G(O) subunit gamma-12 precursor | No |
| *GNG13* | guanine nucleotide-binding protein G(I)/G(S)/G(O) subunit gamma-13 precursor | No |
| *GNG3* | guanine nucleotide-binding protein G(I)/G(S)/G(O) subunit gamma-3 | No |
| *GNG4* | guanine nucleotide-binding protein G(I)/G(S)/G(O) subunit gamma-4 | No |
| *GNB1L* | guanine nucleotide-binding protein subunit beta-like protein 1 | No |
| *GNB2* | guanine nucleotide-binding protein G(I)/G(S)/G(T) subunit beta-2 | No |
| *GNB2L1* | guanine nucleotide-binding protein subunit beta-2-like 1 | No |
| *GNB4* | guanine nucleotide-binding protein subunit beta-4 | No |
| *GNB5* | guanine nucleotide-binding protein subunit beta-5 | No |
| *GLUL* | glutamine synthetase | No |
| *DLG4* | disks large homolog 4 isoform precursor | No |

*GRIA3* was initially included in the gene-set but not taken into account in the association analysis because of the location on the X-chromosome. Other non-included genes were those encoding for the complex guanine nucleotide-binding proteins (G proteins) because of their general involvement in regulating metabolic enzymes, ion channels, and transporters that extends far beyond their involvement in glutamatergic signalling (1). Additionally *GLUL* was not included because it is involved in the synthesis of glutamine instead of glutamate while *DLG4*, which encodes postsynaptic density protein 95, has an important role in regulating multiple processes in addition to glutamatergic signalling such as synaptic plasticity (2).”

**Table S2.** Ingenuity Pathway Analysis (IPA) list of genes included in the GABA-ergic signaling pathway, their functions and inclusion in the gene-set analysis.

| **Gene** | **Function** | **Included** |
| --- | --- | --- |
| *ABAT* | 4-aminobutyrate aminotransferase, mitochondrial precursor | Yes |
| *ADCY1* | adenylate cyclase type 1 | No |
| *ADCY10* | adenylate cyclase type 10 | No |
| *ADCY2* | adenylate cyclase type 2 | No |
| *ADCY3* | adenylate cyclase type 3 | No |
| *ADCY4* | adenylate cyclase type 4 | No |
| *ADCY5* | adenylate cyclase type 5 | No |
| *ADCY6* | adenylate cyclase type 6 | No |
| *ADCY7* | adenylate cyclase type 7 | No |
| *ADCY8* | adenylate cyclase type 8 | No |
| *ADCY9* | adenylate cyclase type 9 | No |
| *ALDH5A1* | succinate-semialdehyde dehydrogenase, mitochondrial isoform | Yes |
| *ALDH9A1* | 4-trimethylaminobutyraldehyde dehydrogenase | Yes |
| *AP1B1* | AP-1 complex subunit beta-1 isoform | No |
| *AP1G2* | AP-1 complex subunit gamma-like 2 isoform | No |
| *AP2A1* | AP-2 complex subunit alpha-1 isoform | No |
| *AP2A2* | AP-2 complex subunit alpha-2 isoform | No |
| *AP2B1* | AP-2 complex subunit beta isoform | No |
| *AP2M1* | AP-2 complex subunit mu isoform | No |
| *AP2S1* | AP-2 complex subunit sigma isoform | No |
| *DNM1* | dynamin-1 isoform | Yes |
| *GABARAP* | gamma-aminobutyric acid receptor-associated protein | Yes |
| *GABBR1* | gamma-aminobutyric acid type B receptor subunit 1 precursor | Yes |
| *GABBR2* | gamma-aminobutyric acid type B receptor subunit 2 precursor | Yes |
| *GABRA1* | gamma-aminobutyric acid receptor subunit alpha-1 precursor | Yes |
| *GABRA2* | gamma-aminobutyric acid receptor subunit alpha-2 precursor | Yes |
| *GABRA3* | gamma-aminobutyric acid receptor subunit alpha-3 precursor | No |
| *GABRA4* | gamma-aminobutyric acid receptor subunit alpha-4 precursor | Yes |
| *GABRA5* | gamma-aminobutyric acid receptor subunit alpha-5 precursor | Yes |
| *GABRA6* | gamma-aminobutyric acid receptor subunit alpha-6 precursor | Yes |
| *GABRB1* | gamma-aminobutyric acid receptor subunit beta-1 precursor | Yes |
| *GABRB2* | gamma-aminobutyric acid receptor subunit beta-2 precursor | Yes |
| *GABRB3* | gamma-aminobutyric acid receptor subunit beta-3 precursor | Yes |
| *GABRD* | gamma-aminobutyric acid receptor subunit delta precursor | Yes |
| *GABRE* | gamma-aminobutyric acid receptor subunit epsilon precursor | No |
| *GABRG1* | gamma-aminobutyric acid receptor subunit gamma-1 precursor | Yes |
| *GABRG2* | gamma-aminobutyric acid receptor subunit gamma-2 precursor | Yes |
| *GABRG3* | gamma-aminobutyric acid receptor subunit gamma-3 precursor | Yes |
| *GABRP* | gamma-aminobutyric acid receptor subunit pi precursor | Yes |
| *GABRQ* | gamma-aminobutyric acid receptor subunit theta precursor | No |
| *GABRR1* | gamma-aminobutyric acid receptor subunit rho-1 precursor | Yes |
| *GABRR2* | gamma-aminobutyric acid receptor subunit rho-2 precursor | Yes |
| *GABRR3* | gamma-aminobutyric acid receptor subunit rho-3 precursor | Yes |
| *GAD1* | glutamate decarboxylase 1 isoform GAD25 | Yes |
| *GAD2* | glutamate decarboxylase 2 | Yes |
| *GNAS* | protein GNAS isoform | No |
| *GPHN* | gephyrin isoform | Yes |
| *GPR37* | prosaposin receptor GPR37 precursor | No |
| *KCNH2* | potassium voltage-gated channel subfamily H member 2 | No |
| *KCNN1* | small conductance calcium-activated potassium channel protein 1 | No |
| *KCNN2* | small conductance calcium-activated potassium channel protein 2 | No |
| *KCNN3* | small conductance calcium-activated potassium channel protein 3 | No |
| *KCNN4* | small conductance calcium-activated potassium channel protein 4 | No |
| *KCNQ2* | potassium voltage-gated channel subfamily KQT member 2 | No |
| *KCNQ3* | potassium voltage-gated channel subfamily KQT member 3 | No |
| *MRAS* | ras-related protein M-Ras precursor | No |
| *NSF* | vesicle-fusing ATPase | Yes |
| *SLC32A1* | vesicular inhibitory amino acid transporter | Yes |
| *SLC6A1* | sodium- and chloride-dependent GABA transporter 1 | Yes |
| *SLC6A11* | sodium- and chloride-dependent GABA transporter 3 | Yes |
| *SLC6A12* | sodium- and chloride-dependent betaine transporter | Yes |
| *SLC6A13* | sodium- and chloride-dependent GABA transporter 2 | Yes |
| *UBQLN1* | ubiquilin-1 isoform | Yes |

*GABRA3*, *GABRE*, and *GABRQ* were initially included in the gene-set but not taken into account in the association analysis because of their location on the X-chromosome. All other genes were not included in the association analysis because of their non-specific role in GABA-ergic signaling. The *ADCY* genes encode the enzymes of the adenyl-cyclase complex, that catalyses the conversion of adenosine triphosphate into cyclic AMP and pyrophosphate, which is important for a large number of biological processes other than GABA signalling (see for instance (3,4)). The AP-1 and AP-2 genes are general transcription factors that are central for regulating the expression and activity of multiple genes in response to stimuli (5). The genes that code for the complexes of small conductance calcium-activated potassium channels and potassium voltage-gated channels are involved in the regulation of action potentials that are generated through the actions of many neurotransmitters. *GNAS* encodes the stimulatory G-protein alpha subunit that is involved in multiple signalling pathways other than GABA signalling. *MRAS* codes for a member of the Ras family of small GTPases and functions as a signal transducer in many ‘generic’ processes such as cell growth and differentiation. Lastly, *GPR37* is a member of the G protein coupled receptor family and has a more general role in regulating neuronal and glial physiology (6).

**Table S3.** Glutamatergic genes and the number of SNPs within the genetic boundaries as well as including a100-kbp flanking region.

| ***Number of SNPs*** | | |
| --- | --- | --- |
| **Glutamatergic genes** | **No flanking** | **100-kbp flanking** |
| *CALM1* | 11 | 125 |
| *CALML5* | *Not captured* | 93 |
| *CAMK4* | 230 | 458 |
| *GLS* | 41 | 172 |
| *GRIA1* | 438 | 600 |
| *GRIA2* | 54 | 205 |
| *GRIA4* | 265 | 427 |
| *GRID1* | 617 | 768 |
| *GRID2* | 746 | 862 |
| *GRIK1* | 381 | 523 |
| *GRIK2* | 658 | 789 |
| *GRIK3* | 90 | 192 |
| *GRIK4* | 276 | 430 |
| *GRIK5* | 6 | 30 |
| *GRIN1* | *Not captured* | 15 |
| *GRIN2A* | 622 | 899 |
| *GRIN2B* | 516 | 707 |
| *GRIN2C* | 7 | 76 |
| *GRIN2D* | 6 | 55 |
| *GRIN3A* | 264 | 443 |
| *GRIN3B* | 1 | 50 |
| *GRINA* | *Not captured* | 9 |
| *GRIP1* | 323 | 475 |
| *GRM1* | 277 | 373 |
| *GRM2* | *Not captured* | 14 |
| *GRM3* | 165 | 254 |
| *GRM4* | 87 | 199 |
| *GRM5* | 531 | 654 |
| *GRM6* | 10 | 172 |
| *GRM7* | 910 | 1106 |
| *GRM8* | 1002 | 1300 |
| *HOMER1* | 57 | 169 |
| *HOMER2* | 52 | 158 |
| *HOMER3* | 1 | 48 |
| *PICK1* | 4 | 111 |
| *SLC17A1* | 67 | 311 |
| *SLC17A2* | 23 | 214 |
| *SLC17A6* | 42 | 134 |
| *SLC17A7* | *Not captured* | 71 |
| *SLC17A8* | 29 | 83 |
| *SLC1A1* | 103 | 312 |
| *SLC1A2* | 187 | 431 |
| *SLC1A3* | 71 | 256 |
| *SLC1A4* | 13 | 156 |
| *SLC1A6* | 20 | 154 |
| *SLC1A7* | 29 | 190 |
| *SLC38A1* | 55 | 193 |

**Table S4.** GABAergic genes and the number of SNPs within the genetic boundaries as well as including a 100-kbp flanking region.

| ***Number of SNPs*** | | |
| --- | --- | --- |
| **GABAergic genes** | **No flanking** | **100-kbp flanking** |
| *ABAT* | 107 | 217 |
| *ALDH5A1* | 33 | 278 |
| *ALDH9A1* | 23 | 199 |
| *DNM1* | 16 | 46 |
| *GABARAP* | *Not captured* | 27 |
| *GABBR1* | 37 | 406 |
| *GABBR2* | 438 | 630 |
| *GABRA1* | 42 | 138 |
| *GABRA2* | 89 | 166 |
| *GABRA4* | 92 | 195 |
| *GABRA5* | 1 | 133 |
| *GABRA6* | 15 | 116 |
| *GABRB1* | 281 | 413 |
| *GABRB2* | 235 | 439 |
| *GABRB3* | 204 | 311 |
| *GABRD* | *Not captured* | 24 |
| *GABRG1* | 71 | 165 |
| *GABRG2* | 60 | 178 |
| *GABRG3* | 397 | 494 |
| *GABRP* | 39 | 204 |
| *GABRR1* | 58 | 185 |
| *GABRR2* | 51 | 216 |
| *GABRR3* | 45 | 260 |
| *GAD1* | 16 | 114 |
| *GAD2* | 77 | 238 |
| *GPHN* | 335 | 484 |
| *NSF* | 22 | 60 |
| *SLC32A1* | *Not captured* | 178 |
| *SLC6A1* | 48 | 212 |
| *SLC6A11* | 123 | 290 |
| *SLC6A12* | 5 | 144 |
| *SLC6A13* | 45 | 168 |
| *UBQLN1* | 42 | 206 |

**Table S5.** Gene-wide associations for all genes in the glutamate and GABA gene-sets when using no flanking region.

|  | ***p*-value** | | | |
| --- | --- | --- | --- | --- |
| **Gene** | **Autism** | **Hyperactivity/ Impulsivity** | **Inattention** | **SSRT** |
| *ABAT* | 0.042434 | 0.31612 | 0.24602 | 0.083319 |
| *ALDH5A1* | 0.7891 | 0.7891 | 0.57201 | 0.72101 |
| *ALDH9A1* | 0.78195 | 0.78195 | 0.86688 | 0.42879 |
| *CALM1* | 0.41265 | 0.146 | 0.57994 | 0.16367 |
| *CAMK4* | 0.19204 | 0.19599 | 0.027561 | 0.28852 |
| *DNM1* | 0.72472 | 0.058169 | 0.10288 | 0.88154 |
| *GABBR1* | 0.5605 | 0.1913 | 0.51159 | 0.16366 |
| *GABBR2* | 0.14454 | 0.39925 | 0.32814 | 0.34161 |
| *GABRA1* | 0.13219 | 0.48784 | 0.67771 | 0.14433 |
| *GABRA2* | 0.031425 | 0.30973 | 0.88334 | 0.89916 |
| *GABRA4* | 0.075928 | 0.24566 | 0.3076 | 0.82527 |
| *GABRA5* | 0.14466 | 0.67609 | 0.50877 |  |
| *GABRA6* | 0.72933 | 0.36216 | 0.083129 | 0.44964 |
| *GABRB1* | 0.20314 | 0.21505 | 0.61332 | 0.12673 |
| *GABRB2* | 0.73361 | 0.79601 | 0.45283 | 0.25295 |
| *GABRB3* | 0.69721 | 0.31011 | 0.5056 | 0.27433 |
| *GABRG1* | 0.22105 | 0.66393 | 0.55156 | 0.12318 |
| *GABRG2* | 0.25422 | 0.42516 | 0.78009 | 0.14987 |
| *GABRG3* | 0.072438 | 0.37534 | 0.37946 |  |
| *GABRP* | 0.55742 | 0.23738 | 0.76089 | 0.97273 |
| *GABRR1* | 0.48654 | 0.8643 | 0.67558 | 0.002711 |
| *GABRR2* | 0.009981 | 0.252 | 0.23001 | 0.27485 |
| *GABRR3* | 0.73867 | 0.34703 | 0.40097 | 0.78523 |
| *GAD1* | 0.089976 | 0.065136 | 0.26791 | 0.27496 |
| *GAD2* | 0.74339 | 0.61291 | 0.84593 | 0.15477 |
| *GLS* | 0.28723 | 0.44964 | 0.61708 | 0.41282 |
| *GPHN* | 0.28728 | 0.51668 | 0.19869 | 0.28563 |
| *GRIA1* | 0.38966 | 0.96735 | 0.24693 | 0.37766 |
| *GRIA2* | 0.74009 | 0.43772 | 0.39849 | 0.60788 |
| *GRIA4* | 0.81754 | 0.3927 | 0.053213 | 0.38408 |
| *GRID1* | 0.21933 | 0.001118 | 0.031218 | 0.19266 |
| *GRID2* | 0.53641 | 0.35917 | 0.37622 | 0.67539 |
| *GRIK1* | 0.18663 | 0.001896 | 0.019578 | 0.076858 |
| *GRIK2* | 0.19569 | 0.67005 | 0.74299 | 0.018062 |
| *GRIK3* | 0.01249 | 0.62494 | 0.40432 | 0.25893 |
| *GRIK4* | 0.68156 | 0.085049 | 0.41807 | 0.93647 |
| *GRIK5* | 0.57929 | 0.79235 | 0.9764 | 0.9381 |
| *GRIN2A* | 0.27403 | 0.029114 | 0.16522 | 0.73415 |
| *GRIN2B* | 0.032321 | 0.003961 | 0.26166 | 0.43811 |
| *GRIN2C* | 0.003591 | 0.4957 | 0.84738 | 0.58948 |
| *GRIN2D* | 0.069227 | 0.1425 | 0.67259 | 0.3701 |
| *GRIN3A* | 0.004415 | 0.92314 | 0.4459 | 0.41118 |
| *GRIN3B* | 0.81048 | 0.29934 | 0.7626 |  |
| *GRIP1* | 0.9239 | 0.27626 | 0.93598 | 0.78818 |
| *GRM1* | 0.60144 | 0.009834 | 0.38045 | 0.17429 |
| *GRM3* | 0.045247 | 0.26292 | 0.85492 | 0.61897 |
| *GRM4* | 0.03293 | 0.055035 | 0.49976 | 0.9425 |
| *GRM5* | 0.046871 | 0.012371 | 0.008675 | 0.96713 |
| *GRM6* | 0.51535 | 0.49984 | 0.13321 | 0.42513 |
| *GRM7* | 0.005357 | 0.15944 | 0.16338 | 0.018695 |
| *GRM8* | 0.41442 | 0.39432 | 0.48456 | 0.62174 |
| *HOMER1* | 0.3697 | 0.021477 | 0.43092 | 0.20995 |
| *HOMER2* | 0.046795 | 0.22071 | 0.59626 | 0.049371 |
| *HOMER3* | 0.090184 | 0.12773 | 0.013209 | 0.97673 |
| *NSF* | 0.96393 | 0.47128 | 0.57376 | 0.65425 |
| *PICK1* | 0.5665 | 0.076444 | 0.79132 | 0.30745 |
| *SLC17A1* | 0.002141 | 0.33591 | 0.61055 | 0.19426 |
| *SLC17A2* | 0.000836 | 0.44624 | 0.44113 | 0.10304 |
| *SLC17A6* | 0.029701 | 0.5957 | 0.34497 | 0.20135 |
| *SLC17A8* | 0.5728 | 0.14257 | 0.10432 | 0.56923 |
| *SLC1A1* | 0.28616 | 0.63595 | 0.25859 | 0.098161 |
| *SLC1A2* | 0.57846 | 0.027963 | 0.5857 | 0.55119 |
| *SLC1A3* | 0.45549 | 0.2894 | 0.77059 | 0.40146 |
| *SLC1A4* | 0.57935 | 0.86356 | 0.29662 | 0.054852 |
| *SLC1A6* | 0.17501 | 0.57592 | 0.2298 | 0.15728 |
| *SLC1A7* | 0.052445 | 0.69654 | 0.11126 | 0.56859 |
| *SLC38A1* | 0.18621 | 0.57486 | 0.043368 | 0.25299 |
| *SLC6A1* | 0.33775 | 0.63242 | 0.26363 | 0.74692 |
| *SLC6A11* | 0.69155 | 0.33054 | 0.65268 | 0.92536 |
| *SLC6A12* | 0.39626 | 0.94597 | 0.57484 | 0.85674 |
| *SLC6A13* | 0.019078 | 0.28369 | 0.19009 | 0.28581 |
| *UBQLN1* | 0.19503 | 0.36137 | 0.87032 | 0.6647 |

None of the genes was significantly associated with any of the phenotypes after correction for multiple comparisons (corrected *p*-value = 0.0003).

**Table S6.** Association result p-values for the discovery and post-hoc tests when only including receptor and transporter genes.

| ***GLUTAMATE*** | Competitive | | |
| --- | --- | --- | --- |
|  | 0 kb | | 100 kb |
| Autism symptom severity | .0898 | | .996 |
| Hyperactivity/impulsivity severity | **.044** | | .997 |
| Inattention severity | .569 | | .768 |
|  |  | |  |
| Inhibition (SSRT) | .118 | | .339 |
|  |  | | |
| ***GABA*** | Competitive | | |
|  | 0 kb | 100 kb | |
| Autism symptom severity | .500 | .721 | |
| Hyperactivity/impulsivity severity | .867 | .643 | |
| Inattention severity | .619 | .092 | |
|  |  |  | |
| Inhibition (SSRT) | .398 | .194 | |

With a more restricted gene-set including only glutamatergic (n=35 when no flanking is used, n=39 when 100kb flanking is used) or GABAergic (n=19, or n=22 respectively) receptor and transporter encoding genes, we found similar results. The significant associations with hyperactivity/impulsivity severity in the larger gene-set, did not survive multiple comparison testing in this more specific analysis. This result suggests that the glutamate and GABA gene-sets associations with ADHD and autism phenotypes are mainly driven by the more restricted gene-set, but that adding additional genes does not only add noise, but further increase the association signal. Thus, including less SNPs decreases the explained variance.

**Figure S1.** Unique genes included in the glutamatergic signaling pathway (Adapted from www.ingenuity.com).

**Figure S2.** Unique genes included in the GABAergic signaling pathway (Adapted from www.ingenuity.com).

# References

1. Neves SR, Ram PT, Iyengar R. G Protein Pathways. Science (80- ). 2002;296(5573):1636–9. 2. Meyer D, Bonhoeffer T, Scheuss V. Balance and stability of synaptic structures during synaptic plasticity. Neuron. 2014;82(2):430–43.

3. Schröder-Lang S, Schwärzel M, Seifert R, Strünker T, Kateriya S, Looser J, et al. Fast manipulation of cellular cAMP level by light in vivo. Nat Methods. 2007;4(1):39–42.

4. Rahman N, Buck J, Levin LR. pH sensing via bicarbonate-regulated “soluble” adenylyl cyclase (sAC). Frontiers in Physiology. 2013;4.

5. Latchman DS. Transcription factors: An overview. Vol. 29, International Journal of Biochemistry and Cell Biology. 1997; 1305–12.

6. Meyer RC, Giddens MM, Schaefer SA, Hall RA. GPR37 and GPR37L1 are receptors for the neuroprotective and glioprotective factors prosaptide and prosaposin. Proc Natl Acad Sci U S A [Internet]. 2013;110(23):9529–34.
