# Supplementary figures and images for: Glutamatergic and GABAergic gene sets in attention-deficit/hyperactivity disorder: association to overlapping traits in ADHD and autism
Source: Transl Psychiatry. 2017 Jan 10;7(1):e999–. doi: 10.1038/tp.2016.273 (PMC5545734; doi:10.1038/tp.2016.273)

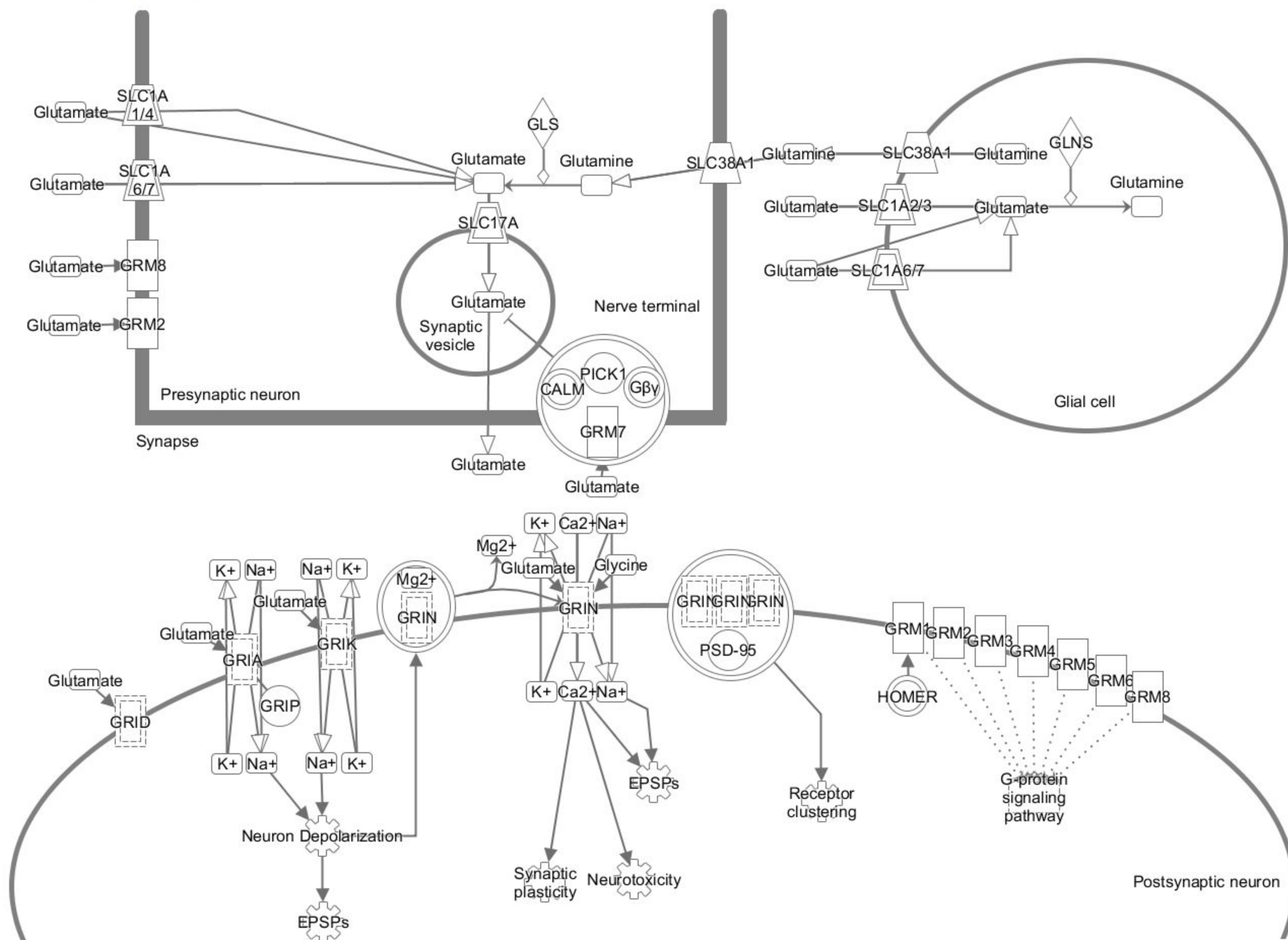

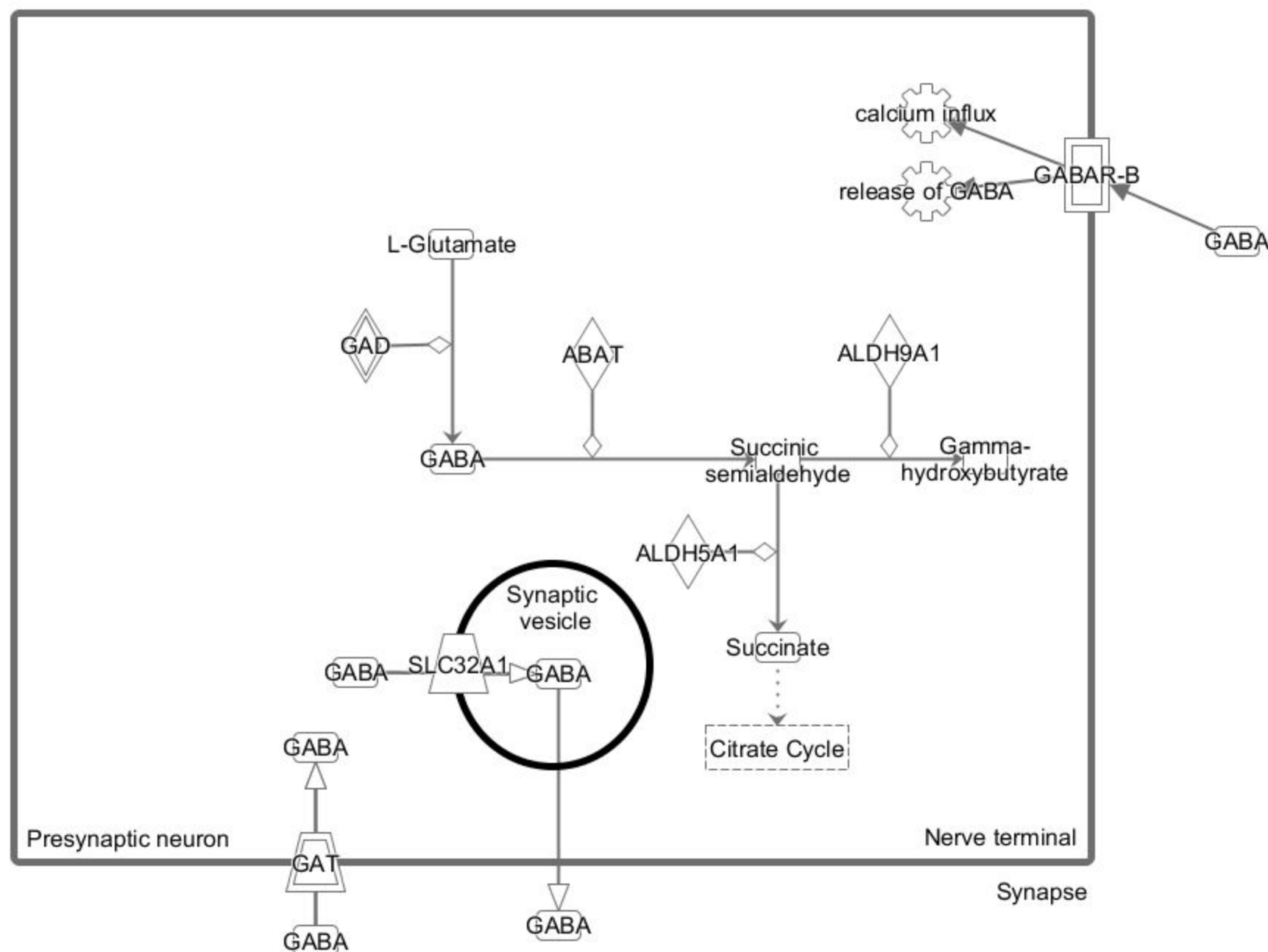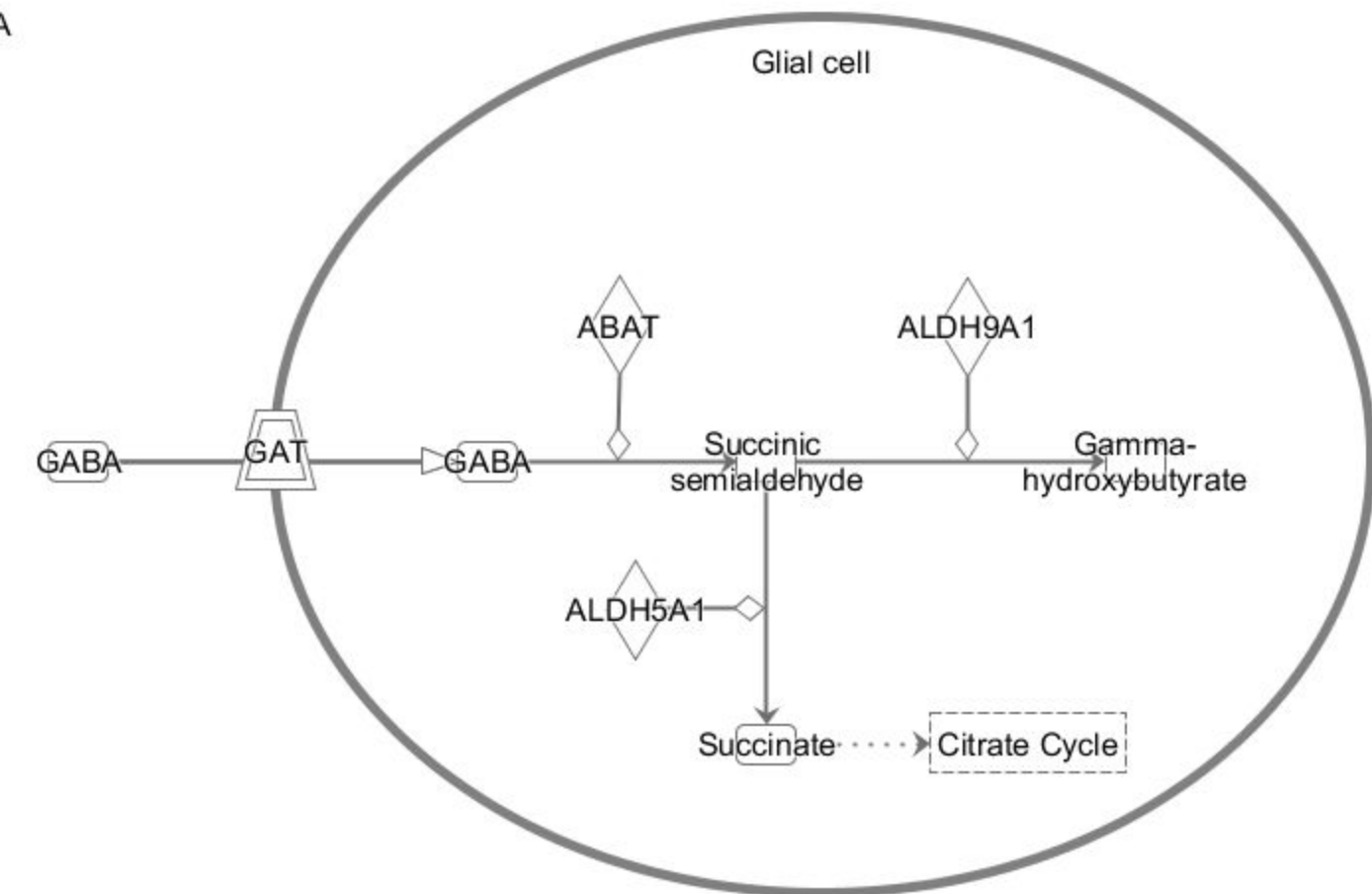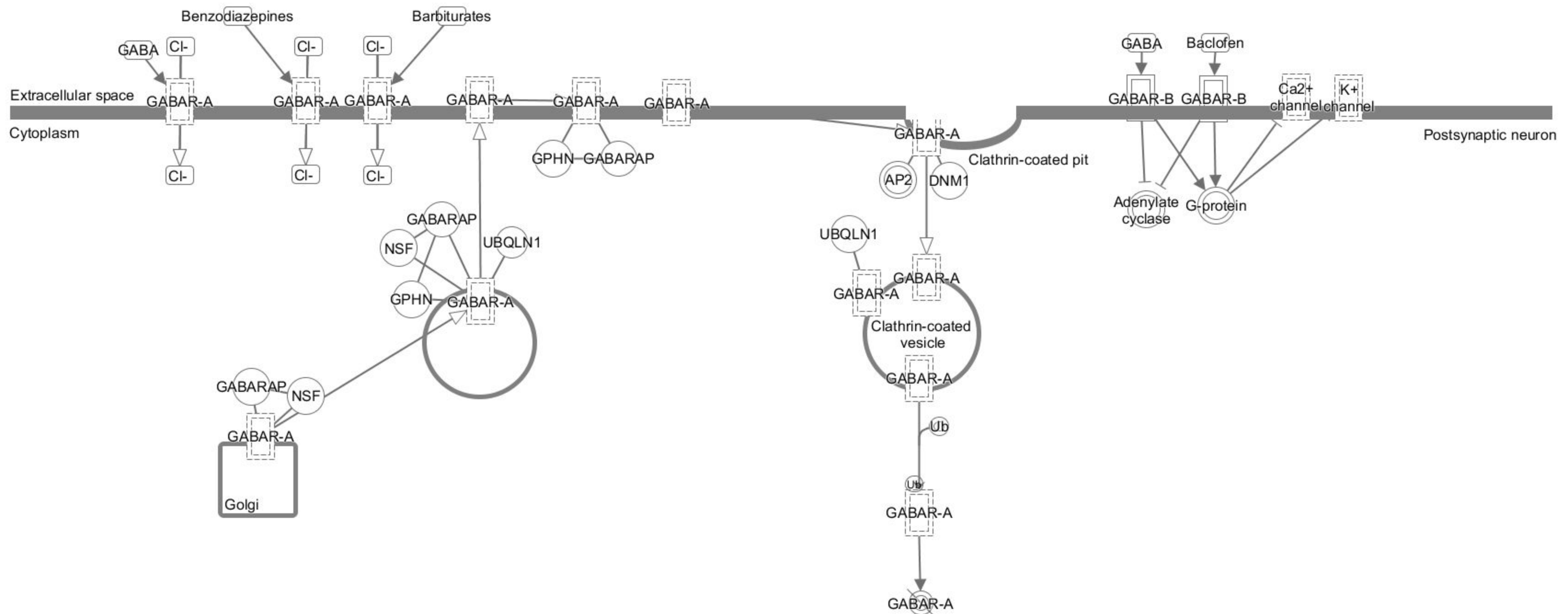

Supplement: Supplementary Figures 1 and 2 [file tp2016273x2.pdf]
